# Molecular Strategy for Survival at a Critical High Temperature in *Eschierichia coli*

**DOI:** 10.1371/journal.pone.0020063

**Published:** 2011-06-10

**Authors:** Masayuki Murata, Hiroko Fujimoto, Kaori Nishimura, Kannikar Charoensuk, Hiroshi Nagamitsu, Satish Raina, Tomoyuki Kosaka, Taku Oshima, Naotake Ogasawara, Mamoru Yamada

**Affiliations:** 1 Applied Molecular Bioscience, Graduate School of Medicine, Yamaguchi University, Ube, Japan; 2 Division of Medical and Biochemical Microbiology, Research Center Borstel, Borstel, Germany; 3 Department of Biological Chemistry, Faculty of Agriculture, Yamaguchi University, Yamaguchi, Japan; 4 Graduate School of Information Science, Nara Institute of Science and Technology, Ikoma, Nara, Japan; University of Kent, United Kingdom

## Abstract

The molecular mechanism supporting survival at a critical high temperature (CHT) in *Escherichia coli* was investigated. Genome-wide screening with a single-gene knockout library provided a list of genes indispensable for growth at 47°C, called thermotolerant genes. Genes for which expression was affected by exposure to CHT were identified by DNA chip analysis. Unexpectedly, the former contents did not overlap with the latter except for *dnaJ* and *dnaK*, indicating that a specific set of non-heat shock genes is required for the organism to survive under such a severe condition. More than half of the mutants of the thermotolerant genes were found to be sensitive to H_2_O_2_ at 30°C, suggesting that the mechanism of thermotolerance partially overlaps with that of oxidative stress resistance. Their encoded enzymes or proteins are related to outer membrane organization, DNA double-strand break repair, tRNA modification, protein quality control, translation control or cell division. DNA chip analyses of essential genes suggest that many of the genes encoding ribosomal proteins are down-regulated at CHT. Bioinformatics analysis and comparison with the genomic information of other microbes suggest that *E. coli* possesses several systems for survival at CHT. This analysis allows us to speculate that a lipopolysaccharide biosynthesis system for outer membrane organization and a sulfur-relay system for tRNA modification have been acquired by horizontal gene transfer.

## Introduction

Responses of *Escherichia coli* to high temperatures have been extensively investigated, though previous studies have mainly focused on the response to a temperature up-shift around 42°C, a response known as a heat shock response (HSR) to induce the expression of a set of proteins, heat-shock proteins (HSPs) [1]. The fact that many HSPs are conserved among species indicates that the actions of HSRs are the fundamentally and physiologically important mechanisms in living organisms [2], [3]. HSPs play crucial roles not only in the rescue or removal of proteins damaged by environmental stresses, including heat stress and salt stress, but also in the intrinsic folding of proteins under normal growth conditions [4].

It has been shown that 384 genes are up-regulated by short-time exposure to a temperature of 43°C as a heat shock in *E. coli* [5], and these genes may be directly or indirectly induced by the treatment. The directly induced genes encode HSPs, including the main cellular chaperone machineries of GroEL and DnaK, ATP-dependent proteases of Lon, HslUV, Clp and FtsH (HflB), periplasmic protease DegP, and other proteins involved in protein folding, refolding, quality control and degradation [6]. HSPs are under complex regulations and are divided into several regulatory groups by their major stimulons [7]. The control of their expression, however, is highly variable among organisms and even among various bacteria [8].

One of the control elements found in Gram-negative bacteria is a heat shock sigma factor that regulates transcription of the major HSPs. HSR in *E. coli* is generally mediated by alternative sigma factors, sigma 32 and sigma 24 [4], [7], [8]. Transcription of the *rpoH* gene for sigma 32 is induced at elevated temperature via the action of sigma 24 [7]. Sigma 24, which is inactive under non-stress conditions by interaction with anti-sigma factor, is activated by misfolding of outer membrane or periplasmic proteins and by stresses including heat shock [9]. Both sigma factors are further regulated at the translation level and or at the posttranslational level. The factor sigma 24 is in part regulated by a cognate small RNA, and sigma 32 synthesis is regulated by structural change of its own mRNA molecules serving as a cellular thermometer and its activity modulated by phosphorylation [10], [11]. Other microorganisms, on the other hand, appear to possess diverged regulatory mechanisms [12].

There is no information on the molecular mechanisms of response to and survival at a critical high temperature (CHT) in organisms, probably due to the limited experimental procedures. Developments of a single-gene knockout library and DNA chip analysis have encouraged us to perform a genome-wide investigation of responses in organisms under extreme conditions. Since several mesophilic bacteria including *E. coli* can grow and survive at high temperatures compared to other mesophilic bacteria, they are assumed to have acquired the potential for thermotolerance during their evolution. In this study, we utilized new procedures for the first time to obtain information on the molecular mechanisms related to thermotolerance in *E. coli* at CHT. Screening of thermosensitive mutants at CHT and informatics analysis of the corresponding genes revealed pathways or factors indispensable for survival at CHT. For essential genes, their possible involvement in the response to CHT was examined by DNA chip analysis. Based on the results, we propose novel molecular mechanisms for survival at CHT in *E. coli*.

## Results and Discussion

### Thermosensitive mutants and thermotolerant genes

In order to identify genes required for survival at CHT in *E. coli*, we screened for thermosensitive mutants from a single-gene knockout library [13], which had been constructed according to the one-step gene disruption method with an *aph* cassette [14] and for which each construct had been confirmed extensively [15]. In the disrupted gene of each mutant strain, the region between the 1^st^ codon and the last 6 codons was displaced with the *aph* cassette, so that most of the coding region of the gene was deleted. Our experiments indicated that the parental strain used for construction of the disrupted library is able to grow at temperatures up to 47°C, this temperature thus being its CHT.

After three successive screening steps of the library, including 3,908 disrupted-mutant strains, 51 strains were found to be sensitive to CHT. Their growth curves at 37°C, 45°C and 46°C were then compared to those of the parental strain (Figure S1). The growth profiles suggest that most mutants selected are significantly sensitive to 46°C and some even to 45°C. Such a disrupted gene responsible for the thermosensitive phenotype was designated as a thermotolerant gene (Table 1 and Table 2).

**Table 1:** Thermotolerant genes identified in this study.

Classification	Sub-classification	Gene	Function	Glc^a^	Mg^2+^ b	H_2_O_2_ c
Energy metabolism	Pyruvate metabolism	*aceE*	pyruvate dehydrogenase, decarboxylase component E1	++		S
(Group A)	Pyruvate metabolism	*aceF*	pyruvate dehydrogenase, dihydrolipoyltransacetylase component E2	++	++	
	Pyruvate metabolism	*lpd*	lipoamide dehydrogenase, E3 component is part of three enzyme complexes	++		
	Pyruvate metabolism	*lipA*	lipoate synthase	++	+	
	Propanate metabolism	*ackA*	acetate kinase A and propionate kinase 2		++	S
	Pentose phosphate pathway	*rpe*	D-ribulose-5-phosphate 3-epimerase	++		S
	Respiratory chain	*cydB*	cytochrome *d* terminal oxidase, subunit II	+	++	
	Respiratory chain	*yhcB*	cytochrome *d* terminal oxidase, subunit III		++	S
Outer membrane	Lipopolysaccharide biosynthesis	*gmhB*	D,D-heptose 1,7-bisphosphate phosphatase		+	
stabilization	Lipopolysaccharide biosynthesis	*lpcA*	D-sedoheptulose 7-phosphate isomerase	+	++	
(Group B)	Lipopolysaccharide biosynthesis	*rfaC*	ADP-heptose:LPS heptosyl transferase I		++	
	Lipopolysaccharide biosynthesis	*rfaD*	ADP-L-glycero-D-mannoheptose-6-epimerase, NAD(P)-binding		++	
	Lipopolysaccharide biosynthesis	*rfaE*	fused heptose 7-phosphate kinase and heptose 1-phosphate adenyltransferase		+	
	Lipopolysaccharide biosynthesis	*rfaF*	ADP-heptose:LPS heptosyltransferase II		+	S
	Lipopolysaccharide biosynthesis	*rfaG*	glucosyltransferase I		+	
	Peptidoglycan-associated lipoprotein	*ydcL*	predicted lipoprotein	+	+	S
	Peptidoglycan-associated lipoprotein	*yfgL*	protein assembly complex, lipoprotein component		++	S
	Peptidoglycan-associated lipoprotein	*ynbE*	predicted lipoprotein	+	+	
	Peptidoglycan-associated lipoprotein	*nlpI*	conserved protein	+	+	
	Peptidoglycan-associated lipoprotein	*ycdO*	conserved protein	+	+	
	Outer membrane integrity	*pal*	peptidoglycan-associated outer membrane lipoprotein		++	
	Outer membrane integrity	*tolQ*	membrane spanning protein in TolA-TolQ-TolR complex		++	
	Outer membrane integrity	*tolR*	membrane spanning protein in TolA-TolQ-TolR complex		++	S
	Outer membrane integrity	*yciM*	conserved hypothetical protein		+	

a According to the data in Figure S4, ratios of growth in the presence of glucose to that in the absence of glucose at 46°C wer estimated. “++” and “+” represent more than 2.0 and 1.5–2.0, respectively.

b According to the data in Figure S4, ratios of growth in the presence of MgCl_2_ to that in the absence of MgCl_2_ at 46°C were estimated. “++” and “+” represent more than 2.0 and 1.5–2.0, respectively.

c According to the data in Figure S4, ratios of growth in the presence of H_2_O_2_ to that in the absence of H_2_O_2_ at 30°C were estimated. “S” represents less than 0.5.

**Table 2:** Thermotolerant genes identified in this study.

Classification	Sub-classification	Gene	Function	Glc^a^	Mg^2+^ b	H_2_O_2_ c
DNA repair	DNA replication & repair, DSBR	*dnaQ*	DNA polymerase III subunit, epsilon		+	S
(Group C)	DNA replication & repair, DSBR	*holC*	DNA polymerase III subunit, chi		++	S
	DNA replication & repair, DSBR	*priA*	primosome factor n'	++	+	S
	DNA repair, DSBR	*ruvA*	component of RuvABC resolvasome, endonuclease	++	++	S
	DNA repair, DSBR	*ruvC*	conserved protein required for cell growth	+	+	S
tRNA modification	tRNA modification	*iscS*	sulfer relay system, cysteine desulfurase			S
(Group D)	tRNA modification	*yheL*	sulfer relay system, predicted intracellular sulfur oxidation protein			S
	tRNA modification	*yheM*	sulfer relay system, predicted intracellular sulfur oxidation protein			S
	tRNA modification	*yheN*	sulfer relay system, predicted intracellular sulfur oxidation protein			S
	tRNA modification	*yhhP*	conserved protein required for cell growth			S
	tRNA modification	*miaA*	delta(2)-isopentenylpyrophosphate tRNA-adenosine transferase		+	S
	tRNA modification	*trmU*	tRNA (5-methylaminomethyl-2-thiouridylate)- methyltransferase			S
	tRNA modification	*truA*	pseudouridylate synthase I	+		
Chaperone/protease	Chaperon system	*dnaJ*	chaperone Hsp40, co-chaperone with DnaK		++	S
(Group E)	Chaperon system	*dnaK*	chaperone Hsp70, co-chaperone with DnaJ			S
	Chaperon system	*degP*	chaperone/serine endoprotease			
	Chaperon regulator	*rseA*	anti-sigma factor		++	S
Translation control	Translation control	*rpmJ*	50S ribosomal subunit L36, related to secY expression			S
(Group F)	Translation control	*rpsF*	30S ribosomal subunit S6, supecifically modified with glutamic acid or phosphate			S
	Translation control	*dksA*	DNA-binding transcriptional regulator or rRNA transcription DnaK suppressor	+	+	S
	Translation control	*smpB*	component of trans-translation process	+	+	S
Cell division	Related to cell division	*xerC*	site-specific tyrosine recombinse involved in chromosome dimer resolution		++	S
(Group G)	Related to cell division	*dedD*	membrane-anchored periplasmic protein involved in separation		++	
	Related to cell division	*envC*	regulator of cell wall hydrolases responsible for cell separation		+	
Others	Membrane transport	*zntA*	zinc/cadmium/mercury/lead-exporting ATPase		+	+
	Membrane transport	*ybgH*	predicted proton-dependent oligopeptide Transporter, POT family	+	++	
	Membrane transpor	*ybhH*	conserved hypothetical protein	++	++	S

a According to the data in Figure S4, ratios of growth in the presence of glucose to that in the absence of glucose at 46°C wer estimated. “++” and “+” represent more than 2.0 and 1.5–2.0, respectively.

b According to the data in Figure S4, ratios of growth in the presence of MgCl_2_ to that in the absence of MgCl_2_ at 46°C were estimated. “++” and “+” represent more than 2.0 and 1.5–2.0, respectively.

c According to the data in Figure S4, ratios of growth in the presence of H_2_O_2_ to that in the absence of H_2_O_2_ at 30°C were estimated. “S” represents less than 0.5.

The gene organization generated by construction of the disrupted mutants might give rise to a polar effect of the inserted *aph* gene on transcription of downstream genes that are intrinsically transcribed by read-through from the promoter or the region upstream of the disrupted gene. Such an organization was found in 42 of the 51 mutants. Sensitivity was not due to a polar effect in 29 of those 42 mutants because disruption of genes just downstream from the disrupted gene by the same method caused no thermosensitive phenotype. The remaining 13 mutants have either an essential gene or a thermotolerant gene as an immediate downstream gene (Figure S2). Their possible polar effects were thus tested by RT-PCR with total RNA prepared from cells exposed to a temperature of 37°C or 47°C (Figure S3). The results suggest that the transcription level of the immediate downstream gene in the mutant was almost the same as that in the parent in all cases except for the cases of mutants of *aceF*, *tolQ*, *dnaK* and *rpsF*. Most of these downstream genes would thus have their own promoters or the transcription level by read-through would be nearly the same as that of the *aph* promoter. However, the transcription levels of *lpd*, *tolR* and *dnaJ* located downstream of *aceF*, *tolQ* and *dnaK*, respectively, were increased and the level of *rpsR* located downstream of *rpsF* was decreased compared to those of the parental strain at both temperatures. Although the expressional alteration of the 4 genes was nearly the same at both temperatures, growth of the corresponding mutant strains at 37°C was not significantly changed from that of the parental strain. Taken together, the results suggest that the thermotolerant phenotype in the 51 mutants is due to disruption of the targeted gene and not due to a polar effect on its downstream genes. Out of the 51 thermotolerant genes, 8 genes, *cydB*, *degP*, *dnaJ*, *dnaK*, *dnaQ*, *nlpI*, *rfaD* and *rfaC*, had been reported as genes supporting growth at a high temperature [16]–[22], and thus we newly identified 43 thermotolerant genes in this organism.

### Effects of supplements and oxidative stress on growth of thermosensitive mutant strains

Since LB was utilized as a medium for the screening of thermosensitive mutants, limitation of carbon source might cause sensitiveness to CHT. We thus examined the effect of glucose as a supplement for growth of the thermosensitive mutant strains (Table 1, Table 2 and Figure S4). We also tested the effect of MgCl_2_ because Mg^2+^ somehow protects against cell damage under stress conditions [23], [24]. The growth of 20 and 37 mutants was improved at CHT by the addition of 0.5% glucose and 20 mM MgCl_2_, respectively. The growth of sixteen mutant strains was improved by supplementation of not only glucose but also MgCl_2_.

Next, the effect of exogenous oxidative stress on the thermosensitive mutant strains was tested since a higher temperature causes more oxidative stress (Noor *et al*, 2009; unpublished data). We exposed thermosensitive mutant strains to 0.5 mM H_2_O_2_ in LB liquid medium at 30°C. Twenty-nine mutants were found to be sensitive to H_2_O_2_ (Table 1, Table 2 and Figure S4), corresponding to approximately 60% of the thermosensitive mutants. Moreover, out of the 10 thermosensitive mutants for which glucose and MgCl_2_ supplementation had no effect, 9 mutants showed sensitivity to H_2_O_2_. These results suggest that the mechanism of thermotolerance at CHT partially overlaps with that of oxidative stress resistance.

### Bioinformatics analysis and classification of thermotolerant genes

To understand the molecular mechanism of *E. coli* survival at CHT, bioinformatics analysis with various public databases including the KEGG PATHWAY database was performed. Out of the 51 thermotolerant genes, 29 genes were successfully mapped on *E. coli* pathways in the KEGG PATHWAY database. Interestingly, many genes were found to be involved in the same metabolic pathway, suggesting that the organism possesses indispensable pathways at CHT. The remaining 19 genes except for 3 unknown genes were extensively analyzed by using the DDBJ or GenBank database. On the basis of results of these analyses and the effects of the supplements, the 51 thermotolerant genes were classified into 7 groups (Table 1 and Table 2).

Group A consists of genes concerned with energy metabolism for production of ATP. The gene products of *aceE*, *aceF*, *lpd* and *ackA* are mapped in the pyruvate metabolism pathway from pyruvate to acetyl CoA [25]–[28] and that of *rpe* is located in the pentose phosphate pathway. *cydB* and *yhcB* encode subunits of cytochrome *d* terminal oxidase, which generates the membrane potential responsible for ATP synthesis [16], [29]. *lipA*, which encodes LipA to produce lipoate required for pyruvate dehydrogenase reaction, also contributes to pyruvate metabolism [30]. Based on the results showing that disrupted mutations of these genes caused a thermosensitive phenotype, we assumed that the cells require more ATP at a higher temperature. This assumption was supported by the finding that the phenotype of most mutants in this group was partially suppressed by the addition of glucose (Table 1 and Figure S4).

Group B consists of genes related to biosynthesis of the cell wall or organization of the outer membrane. The products of *gmhB*, *lpcA(gmhA)*, *rfaC* (*waaC*), *rfaD* (*waaD/htrM*), *rfaE* (*gmhC*), *rfaF* (*waaF*) and *rfaG* (*waaG*) were mapped into the lipopolysaccharide (LPS) biosynthesis pathway [17], [18], [31], [32]. The products of these genes are involved in synthesis of the heptose unit of ADP-L-glycero-D-manno-heptose from sedoheptulose-7phosphate or encode early heptosyl transferases for KDO-lipid A (*rfaC* and *rfaF*) and to further extend the inner core of LPS with glcosyltransferase (*rfaG*). *ydcL*, *yfgL* (*bamB*), *ynbE*, *nlpI* and *ycdO* encode peptidoglycan-associated outer membrane lipoproteins, and the products of *pal*, *tolQ* and *tolR* are components for a complex structure forming a biopolymer transporter [33], [34]. *yciM* encodes a protein possibly required for integrity of the outer membrane [35]. The thermosensitive phenotype caused by disrupted mutants of all of these genes was significantly suppressed by the addition of Mg^2+^ (Table 1 and Figure S4). Since Mg^2+^ is known to stabilize the outer membrane structure by binding extracellularly [36], it is assumed that YdcL, YfgL, YnbE, NlpI, YcdO, Pal, TolQ, TolR and YciM act as components or scaffold proteins of the membrane to maintain outer membrane integrity, especially at a high temperature. Similarly, our data suggest that Mg^2+^ is able to stabilize the outer membrane structure when the LPS biosynthesis pathway becomes defective.

Group C consists of *dnaQ*, *holC*, *priA*, *ruvA* and *ruvC* for DNA double-strand break repair (DSBR) [37]. DnaQ and HolC are epsilon and chi subunits, respectively, of DNA polymerase III [38], [39], which is required for homologous recombination in DSBR [19]. RuvA and RuvC act as DNA helicase and endonuclease, respectively [19], [40], before the replication restart in the DSBR process, and PriA functions as DNA helicase after the replication restart [41]. The requirement of DSBR for survival at CHT suggests that DNA molecules are subjected more to double-strand breaks at a higher temperature. Interestingly, mutants of all members in this group exhibited sensitivity to oxidative stress at 30°C. Therefore, it is thought that there is a strong connection between oxidative stress and DNA double-strand breaks.

Group D includes genes for tRNA modification. Products of *iscS*, *yheL* (*tusB*), *yheM* (*tusC*), *yheN* (*tusD*) and *yhhP* (*tusA*) have been demonstrated to compose the sulfur-relay system [42]–[44]. IscS is a widely distributed cysteine desulfurase that catalyzes desulfuration of L-cysteine by transfer of the sulfur to its active-site cysteine to form a persulfide group (-SSH), being responsible together with YheL, YheM, YheN and YhhP for biosynthesis of the 2-thio modification of 5-methylaminomethyl-2-thiouridine (mnm^5^s^2^U) [43] and five different thio modifications in bacterial tRNAs [45]. IscS also works as a general sulfur donor in various metabolic pathways [46] including biosynthesis of iron-sulfur (Fe-S) cluster [47], thiamine [48], nicotinic acid and branched-chain amino acids [49]. Additionally, *miaA*, *trmU* and *truA* in this group are involved in tRNA modification. The mutations of genes related to sulfer modification cause the phenotype of sensitivity to anti-oxidation stress [44]. Consistently, our study provided evidence that mutants of this group exhibited hypersensitivity to oxidative stress. YheL, YheM, YheN and YhhP, which mainly function in tRNA modification [45], are conserved in thermotolerant bacteria in mesophiles (see Table S3), whereas *iscS*, a general sulfur donor, is widely conserved in mesophiles.

These findings suggest that tRNA modifications presented here are indispensable for growth at CHT.

Group E genes encode chaperones and a protease and thus contribute to the cellular process of regulating heat shock response: *dnaK* and *dnaJ* encode a chaperone and co-chaperone, respectively, for maturation of protein folding or refolding of unfolded proteins [20], [50], and *degP* encodes a chaperone/serine protease located in the periplasm [21]. The indispensability of these genes at CHT suggests that DnaK/DnaJ play a crucial role in dealing with unfolded proteins caused by CHT and that DegP plays an important role in the removal of damaged proteins that have accumulated at such a temperature. *rseA* in this group encodes an anti-sigma factor to keep sigma 24 inactive under non-stress conditions. The thermosensitivity caused by *rseA* disrupted mutation suggests that fine tuning of the intracellular level of active sigma 24 that regulates expression of chaperone or protease genes is somehow crucial for adaptation to the CHT condition. Alternatively, the defective mutant of *rseA* increased sigma 24 activity, which in turn decreased the production of outer membrane proteins via MicA or RybB as a sigma 24 regulon gene [51], resulting in membrane unstability and thermosensitiveness at CHT.

Genes in group F belong to the translation control apparatus. S6 encoded by *rpsF* interacts with the central domain of 16S rRNA and has been demonstrated to play a regulatory rather than a structural role in the ribosome [52]. L36 encoded by *rpmJ* is a component of the 50S subunit of the ribosome, and its disruption decreases the expression of *secY* [53], which encodes a protein-conducting channel in the cytoplasmic membrane. DksA encoded by *dksA* functions as a negative regulator for rRNA genes [54]. Overexpression of DksA has been shown to be a suppressor for a *dnaK* deletion mutation [55] and ensures replication completion by removing transcription roadblocks [56]. SmpB encoded by *smpB* is a component of the trans-translation process and performs rescue of stalled ribosomes with its binding partner, transfer-messenger RNA [57]. These lines of evidence suggest that several constituents in translation pathways are crucial for survival at CHT.

Finally, genes in group G are related to cell division. A *xerC*-encoded protein is a site-specific recombinase [58] and is essential for conversion of chromosome dimers to monomers during cell division. *envC* encodes a component of the cell division machinery that is a direct regulator of the cell wall hydrolase responsible for cell separation that is required for cell division [59]. DedD encoded by *dedD* is a membrane-anchored periplasmic protein involved in septation [60] and has been shown to participate in cytokinesis [61].

The functions of the remaining genes, *ybgH*, *yciM* and *yhhH*, are unknown. Notably, the thermosensitiveness of their mutations was partially suppressed by the addition of Mg^2+^. It is thus likely that their gene products are related to cellular activities similar to those in group B, C or G.

### Possible acquisition of some thermotolerant genes by horizontal gene transfer

Two groups for outer membrane integrity and tRNA modification are almost completely conserved in limited bacterial species with optimal growth at a relatively high temperature (Table S1). Of these group members, genes for the LPS biosynthesis pathway, some lipoproteins and the sulfur-relay system are distributed in very limited bacterial species including *Enterobacteriaceae* (Tables S2 and Table S3). The sulfur-relay system classified in tRNA modification has been demonstrated to modify a few nucleotides of tRNA molecules, contributing to stabilization of their structure, and to be required for survival at an extremely high temperature in *Thermous thermophilus* [62] and it is also conserved in *Thermoanaerobacter tengcongensis* (Table S3). The mature LPS biosynthesis pathway for assembly of the outer membrane consists of many enzyme reactions, which was found to be dispensable at a lower temperature. Interestingly, this pathway is mostly conserved in *Thermodesulfovibrio yellowstonii* and *Thermanaerovibrio acidaminovorans* (Table S2). Enzymes in the LPS biosynthesis and sulfur-relay system in *E. coli* share about 40% sequence identity and about 50% sequence similarity to the corresponding enzymes in thermophilic bacteria. *E. coli* and its closely related bacteria would thus have acquired these genes of the two groups presumably by horizontal gene transfer during their evolution. Since the other five groups are widely conserved not only in thermotolerant mesophilic bacteria but also in other mesophilic bacteria, they would be intrinsically present in *E. coli*. This is consistent with the conserved nature of essentiality of the lipid A part of LPS and essentiality of synthesis of lipid IV_A_ but dispensability of enzymes involved in extension of Kdo_2_-lipid A by various glycosyltransferases. This draws support from Re (*rfaC*) mutants with only tetraacylated lipid A exhibiting a very narrow growth range with ability to grow only under slow growth conditions on minimal medium around 23°C [18], suggesting overall importance of outer membrane integrity at CHT.

### Expressional change caused by heat shock at CHT

None of the thermotolerant genes identified in this study were found to encode HSPs previously identified in *E. coli* except for *dnaJ*, *dnaK*, *degP* and *dnaQ*. To examine whether the thermotolerant genes were up-regulated at CHT or not, we examined transient change in expression of the genomic genes at CHT by DNA chip analysis. The results showed that 42 genes and 111 genes were significantly up-regulated and down-regulated, respectively (Table S4). The up-regulated genes were classified mainly into genes involved in the cellular process, and the down-regulated genes were classified into genes involved in energy metabolism, transport/binding protein and translation. However, none of the thermotolerant genes including *degP* and *dnaQ* as a heat-shock gene were identified as up-regulated genes except for *dnaJ* and *dnaK*. Taken together with data shown above, it is possible that the chaperone systems except for DnaJ/DnaK and GroEL/GroES are not neccesarily involved in thermotolerant mechanisms acquired at CHT. Therefore, it is likely that most products of thermotolerant genes are not HSPs and that the organism possesses a specific set of genes required for survival at CHT.

It is possible that some of the essential genes are crucial for growth at CHT. Such genes, however, could not be examined in this analysis because no disrupted mutants for these genes are available other than the conditional mutants. We thus listed essential genes with significant fluctuation in expression at CHT (Table 3). *groEL* (*groL*) encoding HSP was up-regulated, indicating the possibility that the gene product contributes to survival at CHT. Consistently, it was reported that GroEL appears as a mediator of evolution of extremely heat-resistant *E. coli* cells [63]. On the other hand, 90% of the down-regulated genes were mapped into the translation pathway (Figure S5), encoding for components of ribosomal proteins. It is thus possible that down-regulation of ribosomal genes is one of the strategies for survival at CHT in *E. coli*. Noteworthily, Alix *et al.* reported that ribosome biogenesis in *E. coli* is high temperature-sensitive and DnaK-dependant and predicted that high temperature causes a severe limitation in DnaK/DnaJ to hamper ribosome assembly because heat-induced misfolded proteins would titrate out all the free DnaK/DnaJ [64], [65].

**Table 3:** Essential genes significantly up-regulated and down-regulated at CHT.

Classification^a^	Pathway^a^	Gene
Up-regulated		
Cpn60 chaperonine	RNA degradation	*groEL*
tRNA-Leu	Transfer RNA	*leuU*
Down-regulated		
Lipid metabolism	Fatty acid biosynthesis	*fabG*
Transcription	RNA polymerase	*rpoA*
Translation	16S rRNA processing protein	*rim*
Translation	Translation factors	*fusA*
Translation	Ribosome	*rpsP, rplQ, rpsD, rpsK,*
	*rpsM, rpmC, rplP, rpsC,*	
	*rplV, rpsS, rplB, rplW,*	
	*rplD, rplC, rpsJ, rplJ,*	
	*rplL, rpsR*	

a Classification and Pathway accoding to the KEGG PATHWAY are shown. (http://www.genome.jp/kegg).

### Further consideration on mechanisms for survival at CHT

Two groups of DNA double-strand repair and chaperone/proteinase genes may contribute to endurance against oxidative stress in addition to CHT. Evidence that a higher temperature results in accumulation of more oxidative stress [24] and the finding that mutants of all members in both groups exhibited sensitivity to oxidative stress allow us to speculate that oxidative stress is a main cause of DNA double-strand breaks and of damage to proteins at CHT. Interestingly, oxidative stress is involved in heat-induced cell death in *Saccharomyces cerevisiae* [66], which is supported by the findings that overexpression of catalase and superoxide dismutase genes could increase the degree of thermotolerance and that the thermotolerance is increased under anaerobic conditions. We thus assume that CHT somehow causes intracellular oxidative stress to elicit harmful effects on cells as a secondary stress.

Significant suppression of the thermosensitive phenotype by a defect in the group of energy metabolism (Group A) by the addition of glucose suggests the limitation of energy level at CHT in the organism. The limitation seems to be resolved by alternative pathways that may generate ATP by glucose assimilation. The requirement of ATP at CHT may be consistent with expression of ribosomal genes. Many genes for ribosomal proteins were found to be down-regulated by exposure to CHT, and the disrupted mutant of *dksA* that encodes a negative regulator for rRNA genes became thermosensitive to CHT. These findings and evidence that translation as a ribosomal activity utilizes much energy, up to about 90% of energy consumed in cells [67], suggest that cells manage to reduce energy consumption under a severe condition at CHT. Such saved energy would be utilized for other crucial activities such as repair or degradation of damaged DNA or protein molecules. A smooth translational process at CHT might also save energy, for which S6 and L36 of ribosomal proteins in addition to SmpB may have important functions.

Several strategies for *E. coli* to survive at CHT were discovered. Most of them may also be responsible for other stresses and are conserved even in mesophilic bacteria. Early glycosyltransferases for LPS core biosynthesis for proper outer membrane assembly and permeability barrier function and the sulfur-relay system for tRNA modification might have been acquired for the organism to perform a main task to survive at CHT. Considering the genetic conversion of non-thermotolerant to thermotolerant bacteria, the two strategies might be applicable.

## Materials and Methods

### Materials

Oligonucleotide primers for polymerase chain reaction (PCR) were purchased from FASMAC Co, Ltd (Atsugi, Japan). Other chemicals were all of analytical grade.

### Bacterial strains and growth conditions

Strains used in this study were derivatives of *E. coli* K-12. W3110 (IN (*rrnD-rrnE*), *rph-1*), BW25113 (*rrnB3*, Δ*(lacZ)4787*, *hsdR514*, Δ*(araBAD)567*, Δ*(rhaBAD)568, rph-1*) [14] and mutants of BW25113 in the Keio collection as a single-gene knockout library [13] were grown on plates or in liquid of modified Luria-Bertani (LB) medium (1% Bactotryptone, 0.5% yeast extract, and 0.5% NaCl) at 37°C, 45°C or 47°C for appropriate times.

### Screening of thermosensitive mutants

The Keio collection consisting of 3,908 mutant strains was used for screening. In the 1^st^ screening, mutant strains were grown on LB plates at 30°C overnight. A colony of each strain was patched on LB plates and incubated at 47°C for 48 h to find sensitive strains. The sensitive strains were subjected to the 2^nd^ screening of spotting tests on plates. Cells were cultured in LB medium for 18 h and then diluted with LB medium to adjust turbidity to OD_600_ of 0.5, 0.05 and 0.005. The diluted samples (10 µl) were spotted on LB plates and incubated at 47°C for 48 h. The thermosensitive strains selected by the 2^nd^ screening were subjected to the 3^rd^ screening in liquid culture. After 8-h preculture, cells were diluted to a turbidity corresponding to OD_600_ of 0.1 and inoculated into LB medium at the final OD_600_ of 0.001. Samples were then incubated at 47°C for 18 h under a shaking condition. Thermosensitivity was determined by measuring OD_600_. Thermosensitive strains were defined to be <0.1 at OD_600_. The experiments were performed three times, and the results were confirmed to be reproducible.

### Effects of glucose and MgCl_2_ and sensitivity to H_2_O_2_

To examine effects of supplements, glucose (0.5% (w/v)) or MgCl_2_ (20 mM) was added to the LB liquid culture. After 8-h preculture, cells were diluted to a turbidity corresponding to OD_600_ of 0.1 and inoculated into LB medium with or without the supplement at the final OD_600_ of 0.001. Samples were then incubated at 47°C for 18 h under a shaking condition. After 18 h, turbidity at OD_600_ was measured. To test the sensitivity to oxidative stress, H_2_O_2_ was added to the culture medium at the final concentration of 0.5 mM. After 8-h preculture, cells were diluted to a turbidity corresponding to OD_600_ of 0.1 and inoculated into LB medium with or without H_2_O_2_ at the final OD_600_ of 0.001. Samples were then incubated at 30°C for 8 h under a shaking condition. The experiments were performed three times, and the results were confirmed to be reproducible.

### RT-PCR analysis

Cultures were grown in LB medium at 37°C until the exponential phase, and then the temperature was up-shifted to 47°C and incubation was continued for 8 min. Total RNA was immediately prepared from the heat-stressed cells by the hot phenol method [68]. RT-PCR analysis was performed using an mRNA-selective RT-PCR kit (TAKARA BIO Inc, Otsu, Japan) to examine the expression of immediate downstream genes of disrupted genes as described previously [69]. The primer set used for each gene is shown in Table S5. The RT reaction was carried out at 42°C for 15 min, 85°C for 1 min, 45°C for 1 min and extension at 72°C for 2 min using the two specific primers for each gene. After the completion of 15, 20, 25 and 30 cycles, the PCR products were analyzed by 0.9% agarose gel electrophoresis and stained with ethidium bromide. The relative amounts of RT-PCR products on the gel were compared by measuring the band density after the color of the image taken had been reversed using a model GS-700 Imaging Densitometer (Bio-Rad Laboratories, Inc, Tokyo, Japan) [70].

### Bioinformatics and phylogenetic analyses

Bioinformatics analysis was mainly performed according to the instructions of the KEGG site (http://www.genome.jp/kegg/). Databases of DDBJ (http://www.ddbj.nig.ac.jp) and GenBank (http://www.ncbi.nlm.nih.gov/genbank/) were also used.

### DNA chip analysis

W3110 cultures were grown in LB medium at 37°C until the exponential phase, and then the temperature was up-shifted to 47°C and incubation was continued for 8 min. A control culture was incubated in parallel at 37°C for 8 min. Total RNA was immediately prepared from the heat-stressed cells by the hot phenol method [69]. Preparation of cDNA, fragmentation and the end-labeling of DNA fragments were performed according to the instruction manual from Affymetrix. The ENZO Bioarray terminal labeling kit (Enzo Life Sciences, Inc, New York, USA) was used to end-label DNA fragments. DNA hybridization, data capture and analyses were performed as described in the protocol supplied by Affymetrix and GCOS software (Affymetrix, Inc, California, USA). Two independent experiments were performed and four data sets (two data sets at 37°C: 37°C-1 and 37°C-2, two data sets at 47°C: 47°C-1 and 47°C-2) per gene were obtained. The expression ratio used here indicates the average of the ratios obtained in the two independent experiments. Spots with a significantly lower (<0.50; i.e., a negative fold difference) or higher (2>; i.e., a positive fold difference) fluorescence ratio of the heated sample to the control sample were considered to represent a real significant difference. Physiological function and functional classification of the genes were derived from the Genobase database (http://ecoli.aist-nara.ac.jp/). Array data are accessible through ArrayExpress accession number E-MEXP-3191.

## Supporting Information

Figure S1 **Growth of thermosensitive mutants in LB liquid culture at different temperatures.** Each 51 thermosensitive mutant strain (opened symbols) and the parental strain, BW25113 (closed symbols), were grown in 30 ml LB medium at 37°C (circles), 45°C (squares), or 46°C (triangles). At the times indicated, turbidity at OD_600_ was measured. A, group A; B, group B; C, group C; D, group D; E, group E; F, group F; G, group G; H, others.
(TIF)

Figure S2 **Gene organizations around genes having either an essential gene or a thermotolerant gene as a just downstream gene.** Gene organizations around 13 thermotolerant genes that have either an essential gene or a thermotolerant gene as a just downstream gene are depicted. Black boxes represent identified 13 thermotolerant genes. Grey boxes represent essential or thermotolerant genes. The direction of boxes shows the direction of transcription.
(TIF)

Figure S3 **Testing of possible polar effects by the** ***aph*** **insertion.** Total RNA was prepared from cells cultured at 37°C (A, C) and 47°C (B, D) as described in Materials and Methods. RT-PCR was performed with primers specific for a just downstream gene of each thermotolerant gene to amplify about 500-bp DNA fragments. (A and B) After RT reaction, PCR was performed 15, 20, 25 and 30 cycles and the products were analyzed. (C and D) As a control, each total RNA (10 µg) was submitted to 1.2% agarose gel electrophoresis and staining with ethidium bromide.
(TIF)

Figure S4 **Effects of addition of glucose and MgCl_2_ and sensitivity to H_2_O_2_.** Thermosensitive mutant strains are shown by gene names. Growth conditions are described in Materials and Methods. Black and white columns represent turbidity under the conditions with or without supplements (0.5% glucose (A) or 20 mM MgCl_2_ (B)) or 0.5 mM H_2_O_2_ (C).
(TIF)

Figure S5 **Down-regulated genes for ribosomal proteins.** Systematic analysis of gene function was performed with a database of KEGG PATHWAY. Down-regulated genes for ribosomal proteins were mapped into 6 operons.
(TIF)

Table S1 **Distribution of thermotolerant genes in various bacteria.**
(DOC)

Table S2 **Distribution of thermotolerant genes in group B in various bacteria.**
(DOC)

Table S3 **Distribution of thermotolerant genes in group D in various bacteria.**
(DOC)

Table S4 **Genes significantly up-regulated and down-regulated at CHT.**
(DOC)

Table S5 **RT-PCR primers used in this study.**
(DOC)
