# Supplementary figures and images for: Molecular Strategy for Survival at a Critical High Temperature in Eschierichia coli
Source: PLoS One. 2011 Jun 10;6(6):e20063. doi: 10.1371/journal.pone.0020063 (PMC3112155; doi:10.1371/journal.pone.0020063)

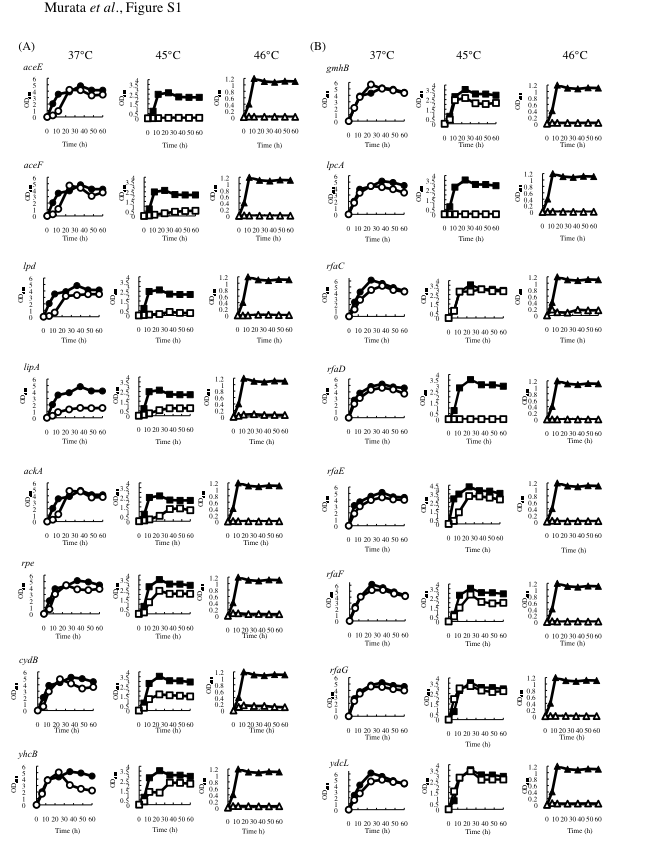

Supplement: Figure S1 — Growth of thermosensitive mutants in LB liquid culture at different temperatures. Each 51 thermosensitive mutant strain (opened symbols) and the parental strain, BW25113 (closed symbols), were grown in 30 ml LB medium at 37°C (circles), 45°C (squares), or 46°C (triangles). At the times indicated, turbidity at OD600 was measured. A, group A; B, group B; C, group C; D, group D; E, group E; F, group F; G, group G; H, others. (TIF) [file pone.0020063.s001.tif]

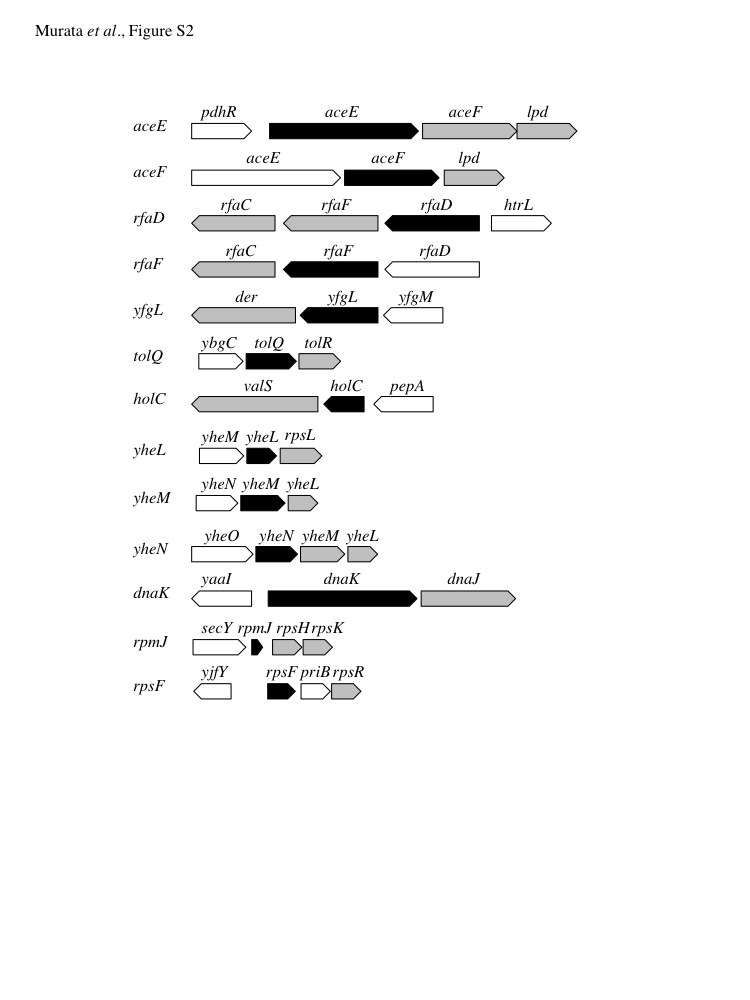

Supplement: Figure S2 — Gene organizations around genes having either an essential gene or a thermotolerant gene as a just downstream gene. Gene organizations around 13 thermotolerant genes that have either an essential gene or a thermotolerant gene as a just downstream gene are depicted. Black boxes represent identified 13 thermotolerant genes. Grey boxes represent essential or thermotolerant genes. The direction of boxes shows the direction of transcription. (TIF) [file pone.0020063.s002.tif]

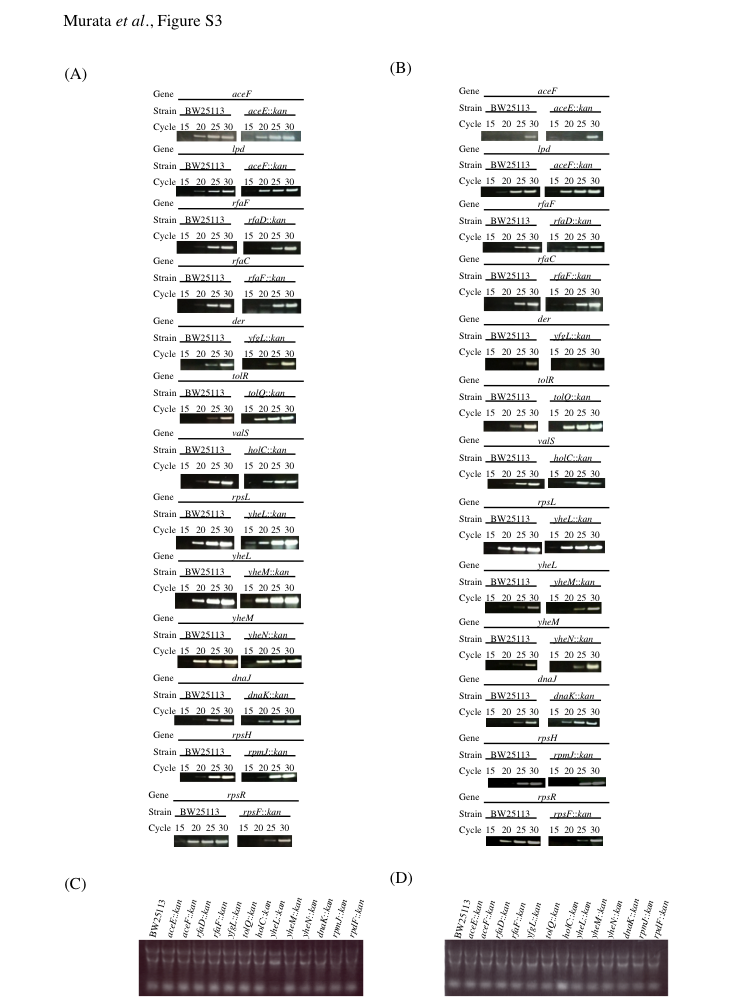

Supplement: Figure S3 — Testing of possible polar effects by the aph insertion. Total RNA was prepared from cells cultured at 37°C (A, C) and 47°C (B, D) as described in Materials and Methods. RT-PCR was performed with primers specific for a just downstream gene of each thermotolerant gene to amplify about 500-bp DNA fragments. (A and B) After RT reaction, PCR was performed 15, 20, 25 and 30 cycles and the products were analyzed. (C and D) As a control, each total RNA (10 µg) was submitted to 1.2% agarose gel electrophoresis and staining with ethidium bromide. (TIF) [file pone.0020063.s003.tif]

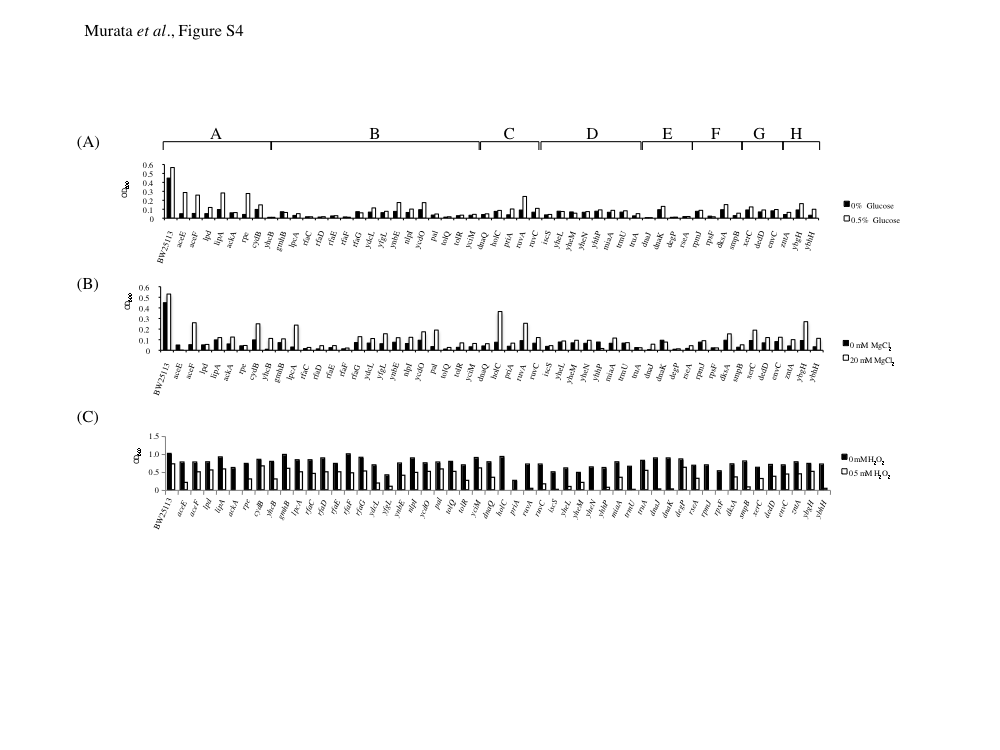

Supplement: Figure S4 — Effects of addition of glucose and MgCl2 and sensitivity to H2O2. Thermosensitive mutant strains are shown by gene names. Growth conditions are described in Materials and Methods. Black and white columns represent turbidity under the conditions with or without supplements (0.5% glucose (A) or 20 mM MgCl2 (B)) or 0.5 mM H2O2 (C). (TIF) [file pone.0020063.s004.tif]

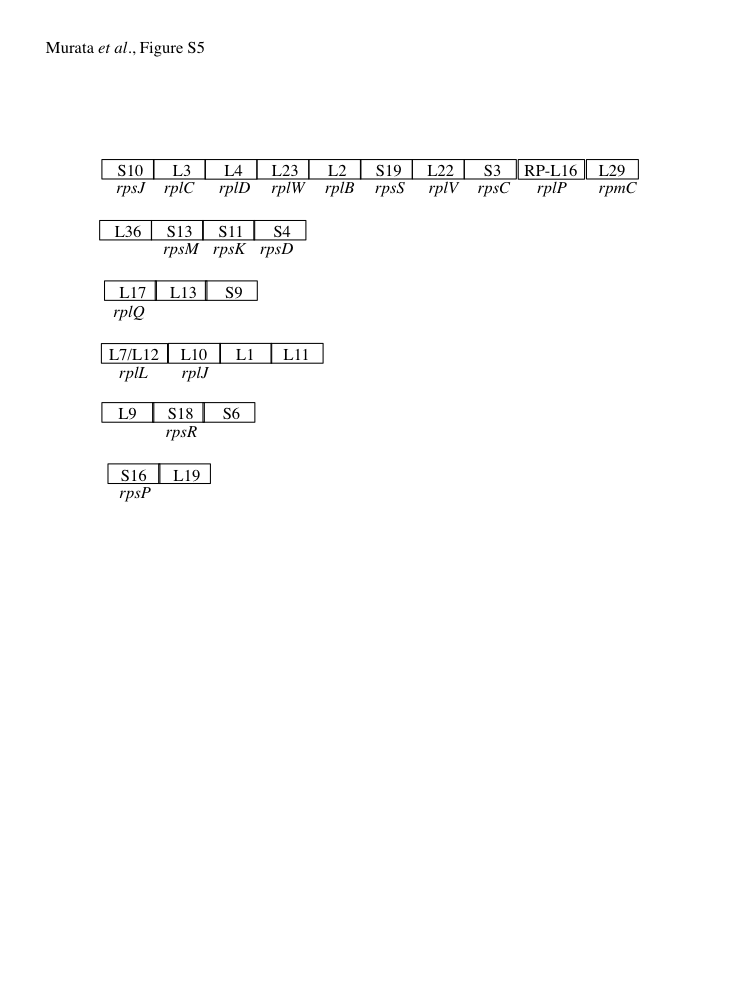

Supplement: Figure S5 — Down-regulated genes for ribosomal proteins. Systematic analysis of gene function was performed with a database of KEGG PATHWAY. Down-regulated genes for ribosomal proteins were mapped into 6 operons. (TIF) [file pone.0020063.s005.tif]
