# Supplementary material for: Molecular Strategy for Survival at a Critical High Temperature in Eschierichia coli
Source: PLoS One. 2011 Jun 10;6(6):e20063. doi: 10.1371/journal.pone.0020063 (PMC3112155; doi:10.1371/journal.pone.0020063)
Supplement: Table S1 — Distribution of thermotolerant genes in various bacteria. (DOC) [file pone.0020063.s006.doc]

**Supplemental Table 1**. Distribution of thermotolerant genes in various bacteria.

| Bacteria^a^ | Optimum  temperature  (ºC) | Energy  metabolism | Outer  membrane  stabilization | DNA  repair | tRNA  modification | Chaperone/  protease | Translation  control | Cell  division |
| --- | --- | --- | --- | --- | --- | --- | --- | --- |
|  |  | (Group A) | (Group B) | (Group C) | (Group D) | (Group E) | (Group F) | (Group G) |
|  |  | 8 genes | 16 genes | 5 genes | 8 genes | 4 genes | 4 genes | 3 genes |
| *Escherichia coli* | 25-39 | 8 | 16 | 5 | 8 | 4 | 4 | 3 |
| *Salmonella enterica* | 30-39 | 8 | 15 | 5 | 8 | 4 | 4 | 3 |
| *Yersinia pestis* | 28-35 | 8 | 14 | 5 | 8 | 4 | 4 | 3 |
| *Shigella flexneri* | 30-35 | 8 | 16 | 5 | 8 | 4 | 4 | 3 |
| *Klebsiella pneumoniae* |  | 8 | 16 | 5 | 8 | 4 | 4 | 3 |
| *Xanthomonas campestris* |  | 6 | 6 | 5 | 3 | 2 | 4 | 2 |
| *Xanthomonas axonopodis* |  | 6 | 6 | 5 | 3 | 2 | 4 | 3 |
| *Vibrio cholerae* | 30-40 | 8 | 10 | 5 | 8 | 4 | 4 | 3 |
| *Pseudomonas aeruginosa* |  | 8 | 13 | 5 | 8 | 4 | 4 | 3 |
| *Pseudomonas putida* | 25-30 | 7 | 12 | 5 | 7 | 4 | 4 | 3 |
| *Pseudomonas syringae* | 25-30 | 8 | 12 | 5 | 8 | 4 | 4 | 3 |
| *Azotobacter vinelandii* |  | 8 | 13 | 5 | 8 | 4 | 4 | 3 |
| *Acinetobacter sp* | 33-35 | 8 | 6 | 5 | 6 | 2 | 4 | 2 |
| *Neisseria meningitidi*s | 35-37 | 6 | 8 | 5 | 4 | 2 | 4 | 2 |
| *Nitosomonas europaea* |  | 6 | 7 | 5 | 4 | 3 | 3 | 3 |
| *Helicobacter pylori* | 30-37 | 2 | 8 | 4 | 4 | 2 | 3 | 1 |
| *Campylobacter jejuni* | 35-37 | 5 | 9 | 4 | 4 | 2 | 4 | 2 |
| *Geobacter sulfurreducens* |  | 6 | 9 | 4 | 4 | 2 | 2 | 2 |
| *Rickettsia prowazekii* |  | 5 | 4 | 5 | 4 | 2 | 4 | 2 |
| *Agrobacterium tumefaciens* | 25-28 | 5 | 4 | 5 | 4 | 2 | 4 | 2 |
| *Rhizobium etli* | 25-30 | 5 | 4 | 5 | 4 | 2 | 4 | 2 |
| *Brucella melitensis* |  | 5 | 4 | 5 | 4 | 2 | 4 | 2 |
| *Rhodopseudomonas palustris* | 30-37 | 6 | 10 | 5 | 4 | 2 | 4 | 2 |
| *Methylobacterium extorquens* | 25-30 | 6 | 4 | 5 | 4 | 2 | 4 | 2 |
| *Caulobacter crescentus* | 25-30 | 6 | 6 | 5 | 4 | 2 | 4 | 2 |
| *Rhodobacter sphaeroides* | 30-34 | 7 | 4 | 5 | 5 | 2 | 4 | 2 |
| *Zymomonas mobilis* | 25-30 | 5 | 4 | 5 | 4 | 2 | 4 | 2 |
| *Gluconacetobacter diazotrophicus* |  | 6 | 6 | 5 | 4 | 2 | 4 | 2 |
| *Acetobacter pasteurianus* |  | 6 | 6 | 5 | 4 | 2 | 3 | 2 |
| *Bacillus subtilis* | 28-30 | 6 | 4 | 2 | 4 | 2 | 3 | 2 |
| *Bacillus cereus* | ~37 | 6 | 2 | 3 | 4 | 2 | 3 | 2 |
| *Bacillus licheniformis* |  | 6 | 2 | 2 | 4 | 2 | 3 | 2 |
| *Staphylococcus aureus* | 30-37 | 6 | 3 | 3 | 4 | 2 | 3 | 2 |
| *Lactococcus lactis* |  | 5 | 2 | 3 | 4 | 2 | 3 | 1 |
| *Streptococcus pyogenes* |  | 4 | 3 | 3 | 4 | 2 | 3 | 1 |
| *Lactobacillus plantarum* | 30-40 | 5 | 3 | 3 | 4 | 2 | 3 | 2 |
| *Clostridium acetobutylicum* |  | 2 | 4 | 3 | 4 | 2 | 3 | 1 |
| *Mycoplasma genitalium* |  | 4 | 0 | 1 | 1 | 2 | 3 | 1 |
| *Mycobacterium tuberculosis* |  | 7 | 3 | 4 | 4 | 2 | 3 | 2 |
| *Corynebacterium glutamicum* |  | 6 | 3 | 4 | 4 | 2 | 2 | 2 |
| *Corynebacterium efficiens* |  | 6 | 3 | 4 | 4 | 2 | 3 | 2 |
| *Streptomyces coelicolor* | 25-35 | 7 | 4 | 4 | 4 | 2 | 4 | 1 |
| *Chlamydia trachomatis* | 37 | 5 | 4 | 4 | 4 | 2 | 4 | 2 |
| *Chlamydophila pneumoniae* | 37 | 5 | 4 | 4 | 4 | 2 | 4 | 2 |
| *Borrelia burgdorferi* |  | 1 | 2 | 2 | 2 | 2 | 3 | 1 |
| *Flavobacterium johnsoniae* |  | 6 | 4 | 4 | 4 | 2 | 4 | 2 |
| *Flavobacterium psychrophilum* |  | 4 | 3 | 4 | 4 | 2 | 3 | 2 |
| *Synechocystis sp* |  | 6 | 3 | 3 | 4 | 2 | 3 | 2 |
| *Chlorobaculum tepidum* |  | 5 | 7 | 4 | 7 | 2 | 3 | 1 |
| *Chlorobium chlorochromatii* | 25-30 | 4 | 8 | 3 | 8 | 2 | 3 | 1 |
| *Deinococcus radiodurans* |  | 6 | 2 | 4 | 4 | 2 | 3 | 1 |
| *Thermotoga maritime* |  | 3 | 2 | 4 | 5 | 2 | 3 | 1 |
| *Archaeoglobus fulgidus* |  | 1 | 2 | 1 | 2 | 0 | 0 | 1 |
| *Pyrococcus horikoshii* |  | 0 | 2 | 0 | 1 | 0 | 0 | 1 |
| *Methylococcus capsulatus* | ~45 | 7 | 8 | 5 | 6 | 3 | 4 | 3 |
| *Methanococcus jannaschii* | 35-40 | 2 | 3 | 0 | 1 | 0 | 0 | 1 |
| *Methanobacterium thermoautotrophicum* | 37-45 | 1 | 2 | 0 | 3 | 1 | 0 | 1 |
| *Halobacterium sp* | 35-50 | 4 | 2 | 0 | 1 | 2 | 0 | 1 |
| *Thermoanaerobacter tengcongensis* | 55-75 | 5 | 3 | 3 | 7 | 2 | 3 | 1 |
| *Thermodesulfovibrio yellowstonii* | 60–65 | 4 | 11 | 4 | 4 | 2 | 3 | 2 |
| *Thermanaerovibrio acidaminovorans* | 60–65 | 1 | 9 | 4 | 4 | 2 | 3 | 2 |

^a^Bacteria shown here are representatives of species of which genomic sequences are available in databases.
