# Supplementary material for: Molecular Strategy for Survival at a Critical High Temperature in Eschierichia coli
Source: PLoS One. 2011 Jun 10;6(6):e20063. doi: 10.1371/journal.pone.0020063 (PMC3112155; doi:10.1371/journal.pone.0020063)
Supplement: Table S2 — Distribution of thermotolerant genes in group B in various bacteria. (DOC) [file pone.0020063.s007.doc]

**Supplemental Table 2**. Distribution of thermotolerant genes in group B in various bacteria.

| Bacteria^a^ | *gmhB* | *lpcA* | *rfaC* | *rfaD* | *rfaE* | *rfaF* | *rfaG* | *ydcL* | *yfgL* | *ynbE* | *nlpI* | *ycdO* | *pal* | *tolQ* | *tolR* | *yciM* |
| --- | --- | --- | --- | --- | --- | --- | --- | --- | --- | --- | --- | --- | --- | --- | --- | --- |
| *Escherichia coli* | o | o | o | o | o | o | o | o | o | o | o | o | o | o | o | o |
| *Salmonella enterica* | o | o | o | o | o | o | o | o | o | o | o | x | o | o | o | o |
| *Yersinia pestis* | o | o | o | o | o | o | x | x | o | o | o | o | o | o | o | o |
| *Shigella flexneri* | o | o | o | o | o | o | o | o | o | o | o | o | o | o | o | o |
| *Klebsiella pneumoniae* | o | o | o | o | o | o | o | o | o | o | o | o | o | o | o | o |
| *Xanthomonas campestris* | x | x | x | x | x | x | x | o | o | x | x | x | o | o | o | o |
| *Xanthomonas axonopodis* | x | x | x | x | x | x | x | o | o | x | x | x | o | o | o | o |
| *Vibrio cholerae* | o | o | x | o | x | o | x | x | o | x | o | x | o | o | o | o |
| *Pseudomonas aeruginosa* | o | o | o | o | o | o | o | x | o | o | x | x | o | o | o | o |
| *Pseudomonas putida* | o | o | o | x | o | o | o | o | o | o | x | x | x | o | o | o |
| *Pseudomonas syringae* | o | o | o | x | o | o | o | x | o | x | x | o | o | o | o | o |
| *Azotobacter vinelandii* | o | o | o | x | o | o | o | o | o | o | x | x | o | o | o | o |
| *Acinetobacter sp* | x | x | x | x | x | x | x | o | o | x | x | x | o | o | o | o |
| *Neisseria meningitidi*s | o | o | o | o | x | o | x | x | o | x | x | o | x | x | x | o |
| *Nitosomonas europaea* | o | x | x | x | x | x | x | o | o | x | x | x | o | o | o | o |
| *Helicobacter pylori* | o | o | o | o | o | o | x | x | x | x | x | x | o | x | x | o |
| *Campylobacter jejuni* | o | o | o | o | o | o | x | x | o | x | x | x | o | x | x | o |
| *Geobacter sulfurreducens* | o | o | o | x | o | o | x | x | o | x | x | x | o | o | x | o |
| *Rickettsia prowazekii* | x | x | x | x | x | x | x | x | o | x | x | x | o | o | x | o |
| *Agrobacterium tumefaciens* | x | x | x | x | x | x | x | x | o | x | x | x | o | o | x | o |
| *Rhizobium etli* | x | x | x | x | x | x | x | x | o | x | x | x | o | o | x | o |
| *Brucella melitensis* | x | x | x | x | x | x | x | x | o | x | x | x | o | o | x | o |
| *Rhodopseudomonas palustris* | o | o | o | o | o | o | o | x | o | x | x | x | x | o | x | o |
| Bacteria^a^ | *gmhB* | *lpcA* | *rfaC* | *rfaD* | *rfaE* | *rfaF* | *rfaG* | *ydcL* | *yfgL* | *ynbE* | *nlpI* | *ycdO* | *pal* | *tolQ* | *tolR* | *yciM* |
| *Methylobacterium extorquens* | x | x | x | x | x | x | x | x | o | x | x | x | o | o | x | o |
| *Caulobacter crescentus* | x | x | x | o | o | x | x | x | o | x | x | x | o | o | x | o |
| *Rhodobacter sphaeroides* | x | x | x | x | x | x | x | x | o | x | x | x | o | o | x | o |
| *Zymomonas mobilis* | x | x | x | x | x | x | x | x | o | o | x | x | o | x | x | o |
| *Gluconacetobacter diazotrophicus* | x | x | x | o | o | x | x | x | o | x | x | x | o | o | x | o |
| *Acetobacter pasteurianus* | x | x | x | o | o | x | x | x | o | x | x | x | o | o | x | o |
| *Bacillus subtilis* | x | x | x | x | x | x | x | o | o | x | x | o | x | x | x | o |
| *Bacillus cereus* | x | x | x | x | x | x | x | x | o | x | x | x | x | x | x | o |
| *Bacillus licheniformis* | x | x | x | x | x | x | x | x | o | x | x | x | x | x | x | o |
| *Staphylococcus aureus* | x | x | x | x | x | x | x | o | o | x | x | x | x | x | x | o |
| *Lactococcus lactis* | x | x | x | x | x | x | x | x | o | x | x | x | x | x | x | o |
| *Streptococcus pyogenes* | x | x | x | x | x | x | x | o | o | x | x | x | x | x | x | o |
| *Lactobacillus plantarum* | x | x | x | x | x | x | x | o | o | x | x | x | x | x | x | o |
| *Clostridium acetobutylicum* | o | o | x | x | x | x | x | x | o | x | x | x | x | x | x | o |
| *Mycoplasma genitalium* | x | x | x | x | x | x | x | x | x | x | x | x | x | x | x | x |
| *Mycobacterium tuberculosis* | o | o | x | x | x | x | x | x | o | x | x | x | x | x | x | x |
| *Corynebacterium glutamicum* | x | x | x | x | x | x | x | o | o | x | x | x | x | x | x | o |
| *Corynebacterium efficiens* | x | x | x | x | x | x | x | x | o | x | x | x | x | x | x | o |
| *Streptomyces coelicolor* | x | o | x | x | x | x | x | x | o | x | x | o | x | x | x | o |
| *Chlamydia trachomatis* | x | x | x | x | x | x | x | x | o | x | x | o | o | x | x | o |
| *Chlamydophila pneumoniae* | x | x | x | x | x | x | x | x | o | x | x | o | o | x | x | o |
| *Borrelia burgdorferi* | x | x | x | x | x | x | x | x | o | x | x | x | x | x | x | o |
| *Flavobacterium johnsoniae* | x | x | x | x | x | o | x | x | o | x | x | x | o | x | x | o |
| *Flavobacterium psychrophilum* | x | x | x | x | x | x | x | x | o | x | x | x | o | x | x | o |
| Bacteria^a^ | *gmhB* | *lpcA* | *rfaC* | *rfaD* | *rfaE* | *rfaF* | *rfaG* | *ydcL* | *yfgL* | *ynbE* | *nlpI* | *ycdO* | *pal* | *tolQ* | *tolR* | o |
| *Synechocystis sp* | x | o | x | x | x | x | x | x | o | x | x | x | x | x | x | o |
| *Chlorobaculum tepidum* | o | o | x | o | x | x | x | x | x | o | x | x | o | o | x | o |
| *Chlorobium chlorochromatii* | o | o | x | o | x | x | x | x | o | o | x | x | o | o | x | o |
| *Deinococcus radiodurans* | x | x | x | x | x | x | x | x | o | x | x | x | x | x | x | o |
| *Thermotoga maritime* | x | x | x | x | x | x | x | x | o | x | x | x | x | x | x | o |
| *Archaeoglobus fulgidus* | x | x | x | x | x | x | x | x | o | x | x | x | x | x | x | o |
| *Pyrococcus horikoshii* | x | x | x | x | x | x | x | x | o | x | x | x | x | x | x | o |
| *Methylococcus capsulatus* | o | o | x | x | o | x | x | x | o | x | x | x | o | o | o | o |
| *Methanococcus jannaschii* | x | o | x | x | x | x | x | x | x | x | x | x | x | x | x | o |
| *Methanobacterium thermoautotrophicum* | x | x | x | x | x | x | x | x | o | x | x | x | x | x | x | o |
| *Halobacterium sp* | x | x | x | x | x | x | x | x | o | x | x | x | x | x | x | o |
| *Thermoanaerobacter tengcongensis* | x | o | x | x | x | x | x | x | o | x | x | x | x | x | x | o |
| *Thermodesulfovibrio yellowstonii* | x | o | o | o | x | x | o | x | o | x | o | o | o | o | o | o |
| *Thermanaerovibrio acidaminovorans* | o | o | o | x | o | x | x | x | x | x | x | o | o | o | o | o |
| ^a^Bacteria shown here are representatives of species of which genomic sequences are available in databases.  “o” and “x” represent the presence and absence of themotorelant genes in group B, respectively. | | | | | | | | | | | | | | | | |
