# Supplementary material for: Molecular Strategy for Survival at a Critical High Temperature in Eschierichia coli
Source: PLoS One. 2011 Jun 10;6(6):e20063. doi: 10.1371/journal.pone.0020063 (PMC3112155; doi:10.1371/journal.pone.0020063)
Supplement: Table S3 — Distribution of thermotolerant genes in group D in various bacteria. (DOC) [file pone.0020063.s008.doc]

**Supplemental Table 3**. Distribution of thermotolerant genes in group D in various bacteria.

| Bacteria^a^ | *iscS* | *yheL* | *yheM* | *yheN* | *yhhP* | *miaA* | *trmU* | *truA* |
| --- | --- | --- | --- | --- | --- | --- | --- | --- |
| *Escherichia coli* | o | o | o | o | o | o | o | o |
| *Salmonella enterica* | o | o | o | o | o | o | o | o |
| *Yersinia pestis* | o | o | o | o | o | o | o | o |
| *Shigella flexneri* | o | o | o | o | o | o | o | o |
| *Klebsiella pneumoniae* | o | o | o | o | o | o | o | o |
| *Xanthomonas campestris* | x | x | x | x | x | o | o | o |
| *Xanthomonas axonopodis* | x | x | x | x | x | o | o | o |
| *Vibrio cholerae* | o | o | o | o | o | o | o | o |
| *Pseudomonas aeruginosa* | o | o | o | o | o | o | o | o |
| *Pseudomonas putida* | o | o | o | o | o | o | o | x |
| *Pseudomonas syringae* | o | o | o | o | o | o | o | o |
| *Azotobacter vinelandii* | o | o | o | o | o | o | o | o |
| *Acinetobacter sp* | o | x | x | o | o | o | o | o |
| *Neisseria meningitidi*s | o | x | x | x | x | o | o | o |
| *Nitosomonas europaea* | o | x | x | x | x | o | o | o |
| *Helicobacter pylori* | o | x | x | x | x | o | o | o |
| *Campylobacter jejuni* | o | x | x | x | x | o | o | o |
| *Geobacter sulfurreducens* | o | x | x | x | x | o | o | o |
| *Rickettsia prowazekii* | o | x | x | x | x | o | o | o |
| *Agrobacterium tumefaciens* | o | x | x | x | x | o | o | o |
| *Rhizobium etli* | o | x | x | x | x | o | o | o |
| *Brucella melitensis* | o | x | x | x | x | o | o | o |
| *Rhodopseudomonas palustris* | o | x | x | x | x | o | o | o |
| *Methylobacterium extorquens* | o | x | x | x | x | o | o | o |
| *Caulobacter crescentus* | o | x | x | x | x | o | o | o |
| *Rhodobacter sphaeroides* | o | x | x | x | o | o | o | o |
| *Zymomonas mobilis* | o | x | x | x | x | o | o | o |
| *Gluconacetobacter diazotrophicus* | o | x | x | x | x | o | o | o |
| *Acetobacter pasteurianu* | o | x | x | x | x | o | o | o |
| Bacteria^a^ | *iscS* | *yheL* | *yheM* | *yheN* | *yhhP* | *miaA* | *trmU* | *truA* |
| *Bacillus subtilis* | o | x | x | x | x | o | o | o |
| *Bacillus cereus* | o | x | x | x | x | o | o | o |
| *Bacillus licheniformis* | o | x | x | x | x | o | o | o |
| *Staphylococcus aureus* | o | x | x | x | x | o | o | o |
| *Lactococcus lactis* | o | x | x | x | x | o | o | o |
| *Streptococcus pyogenes* | o | x | x | x | x | o | o | o |
| *Lactobacillus plantarum* | o | x | x | x | x | o | o | o |
| *Clostridium acetobutylicum* | o | x | x | x | x | o | o | o |
| *Mycoplasma genitalium* | x | x | x | x | x | x | x | o |
| *Mycobacterium tuberculosis* | o | x | x | x | x | o | o | o |
| *Corynebacterium glutamicum* | o | x | x | x | x | o | o | o |
| *Corynebacterium efficiens* | o | x | x | x | x | o | o | o |
| *Streptomyces coelicolor* | o | x | x | x | x | o | o | o |
| *Chlamydia trachomatis* | o | x | x | x | x | o | o | o |
| *Chlamydophila pneumoniae* | o | x | x | x | x | o | o | o |
| *Borrelia burgdorferi* | o | x | x | x | x | x | x | o |
| *Flavobacterium johnsoniae* | o | x | x | x | x | o | o | o |
| *Flavobacterium psychrophilum* | o | x | x | x | x | o | o | o |
| *Synechocystis sp* | o | x | x | x | x | o | o | o |
| *Chlorobaculum tepidum* | o | x | o | o | o | o | o | o |
| *Chlorobium chlorochromati* | o | o | o | o | o | o | o | o |
| *Deinococcus radiodurans* | o | x | x | x | x | o | o | o |
| *Thermotoga maritime* | o | o | x | x | x | o | o | o |
| *Archaeoglobus fulgidus* | o | x | x | x | x | x | x | o |
| *Pyrococcus horikoshii* | x | x | x | x | x | x | x | o |
| *Methylococcus capsulatus* | o | o | x | o | x | o | o | o |
| *Methanococcus jannaschii* | x | x | x | x | x | x | x | o |
| *Methanobacterium thermoautotrophicum* | x | o | o | x | x | x | x | o |
| *Halobacterium sp* | x | x | x | x | x | x | x | o |
| *Thermoanaerobacter tengcongensis* | o | o | o | o | x | o | o | o |
| *Thermodesulfovibrio yellowstonii* | o | x | x | x | x | o | o | o |
| Bacteria^a^ | *iscS* | *yheL* | *yheM* | *yheN* | *yhhP* | *miaA* | *trmU* | *truA* |
| *Thermanaerovibrio acidaminovorans* | o | x | x | x | x | o | o | o |

^a^Bacteria shown here are representatives of species of which genomic sequences are available in databases.

“o” and “x” represent the presence and absence of themotorelant genes in group D, respectively.
