# Supplementary material for: Molecular Strategy for Survival at a Critical High Temperature in Eschierichia coli
Source: PLoS One. 2011 Jun 10;6(6):e20063. doi: 10.1371/journal.pone.0020063 (PMC3112155; doi:10.1371/journal.pone.0020063)
Supplement: Table S4 — Genes significantly up-regulated and down-regulated at CHT. (DOC) [file pone.0020063.s009.doc]

**Supplemental Table 4**. Genes significantly up-regulated and down-regulated at CHT.

| Classification | Number of  classified genes | Up-regulated* | Down-regulated* |
| --- | --- | --- | --- |
| 1) Amino acid metabolism | 136 (5) | 0 (0) | 3 (0) |
| 2) Biosynthesis of cofactors, prosthetic groups, carriers | 127 (28) | 1 (0) | 0 (0) |
| 3) Cell envelope | 171 (16) | 0 (0) | 5 (0) |
| 4) Cellular process | 102 (63) | 6 (1) | 5 (0) |
| 5) Central intermediately metabolism | 153 (8) | 2 (0) | 8 (0) |
| 6) Energy metabolism | 358 (7) | 2 (0) | 22 (0) |
| 7) Fatty acid/Phospholipid metabolism | 40 (22) | 0 (0) | 2 (1) |
| 8) Nucleotide metabolism | 113 (0) | 0 (0) | 4 (0) |
| 9) Regulatory functions | 107 (1) | 1 (0) | 4 (0) |
| 10) Replication | 79 (23) | 0 (0) | 1 (0) |
| 11) Transport/binding protein | 76 (1) | 0 (0) | 12 (0) |
| 12) Translation | 354 (101) | 4 (1) | 23 (20) |
| 13) Transcription | 77 (13) | 1 (0) | 1(1) |
| 14) Other categories | 336 (14) | 3 (0) | 5 (0) |
| 15) Hypothetical | 2269 (10) | 20 (0) | 16 (0) |
| Total number of genes | 4498 (312) | 42 (2) | 111 (22) |

*Genes with expression ratios of more than twofold, or less than 25%, are shown as significantly up-regulated

and down-regulated, respectively. *Digits inside parentheses represent the number of essential genes.
