# Supplementary material for: Molecular Strategy for Survival at a Critical High Temperature in Eschierichia coli
Source: PLoS One. 2011 Jun 10;6(6):e20063. doi: 10.1371/journal.pone.0020063 (PMC3112155; doi:10.1371/journal.pone.0020063)
Supplement: Table S5 — RT-PCR primers used in this study. (DOC) [file pone.0020063.s010.doc]

**Supplemental Table 5.** RT-PCR primers used in this study.

| Name | Sequence |
| --- | --- |
| aceF-5’ | 5’-GAGATCCTGGTCAAAGT-3’ |
| aceF-3’ | 5’-GCCAGCAAACGGAGCCG-3’ |
| lpd-5’ | 5’-AACTCAGGTCGTGGTAC-3’ |
| lpd-3’ | 5’-AACGGCAGTTGGATCGG-3’ |
| rfaC-5’ | 5’-CGATGGGCGATGTTCTC-3’ |
| rfaC-3’ | 5’-CATCTGTAGGCAGGTTC-3’ |
| rfaF-5’ | 5’-ACATGATGATGTCGCAA-3’ |
| rfaF-3’ | 5’-TTGTTATGGCCGCAGTT-3’ |
| der-5’ | 5’-GTCTAACTCGCACCCGA-3’ |
| der-3’ | 5’-CCGTTCTCTTCCGCTTC-3’ |
| tolR-5' | 5’-GAGGTCGTCGCGATCTCA-3’ |
| tolR-3' | 5’-CGGTTGGTTTAATGACGCA-3’ |
| valS-5’ | 5’-AAGATATCGAACAGCCG-3’ |
| valS-3’ | 5’-TTCACCGCATTGGACAG-3’ |
| rpsL-5’ | 5’-CAGTTAACCAGCTGGTA-3’ |
| rpsL-3’ | 5’-CTTCACGCCATACTTGG-3’ |
| yheL-5’ | 5’-CACATTACATCGCTCAC-3’ |
| yheL-3’ | 5’-TCACCAGGCCATCTGGC-3’ |
| yheM-5’ | 5’-CTACTGCACCTCATGGT-3’ |
| yheM-3’ | 5’-TCGTAGTTGGCGAGTTC-3’ |
| yheN-5’ | 5’-TTGCCATCGTGGTGACC-3’ |
| yheN-3’ | 5’-GAGCGAGGCTTCCGCCA-3’ |
| dnaJ-5’ | 5’-CAGCGGAAGAGCGTGAA-3’ |
| dnaJ-3’ | 5’-GAAGAATCCCTGGCGCA-3’ |
| rpsM-5’ | 5’-CCGTATAGCAGGCATTA-3’ |
| rpsM-3’ | 5’-TTGATCGGTTTGCGCGG-3’ |
| rpsK-5' | 5’-GGCAAAGGCACCAATTCG-3’ |
| rpsK-3' | 5’-TAGTGATGCGGAAACCTG-3’ |
| rpsR-5' | 5’-ACGTTATTTCCGTCGTCG-3’ |
| rpsR-3' | 5’-GATCAGTGTACGGCAGCA-3’ |
